# Practical Things in Dental Practice

**Published:** 1897-12

**Authors:** J. G. Templeton

**Affiliations:** Pittsburg, Pa.


ARTICLE IX.

### PRACTICAL THINGS IN DENTAL PRACTICE.

BY J. G. TEMPLETON, A. M., D. D. S., PITTSBURG, PA.

To protect the pulp from thermal changes, thoroughly dry the cavity with absolute alcohol, next coat the cavity with varnish made by dissolving common resin in chloroform, then cut from a thin piece of asbestos felt a piece just large enough to cover the bottom of the cavity, and moisten with wood-creosote or campho-phenique, and cover one side with a mixture of oxid of zinc, iodol, and vaseline or aboline, and place it over the bottom of the cavity, and a filling can be inserted that will not convey heat or cold to the pulp.

The old-fashioned bur drill and excavator should be used more than they are in the preparation of cavities instead of relying entirely on the burring engine.

The engine and the sharp bur may be satisfactory to the operator, but very much less so to the patient.

To make moisture tight gutta-percha fillings: Dry the cavity well, place in it a pellet of cotton saturated with absolute alcohol, remove the cotton and with a warm air syringe evaporate the alcohol, varnish the cavity with a solution of common resin in chloroform, warm the gutta-percha and pack with a cold instrument; heat a thin-bladed instrument and pare off the surplus gutta-percha; any further trimming or polishing required may be done with oil of cajeput.

To protect from thermal changes, particularly in deep cavities where the pulp is not quite exposed, first dry with bibulous paper, then apply on a small pellet of cotton absolute alcohol, which has a strong affinity for any moisture that may be left in the cavity or open ends of the tubuli. When the cotton is removed evaporation takes place rapidly, leaving the cavity dry. Now varnish inside of cavity to near the margin with a solution of common resin, in chloroform, or of gum sandarac dissolved in sulfuric ether; then take a small piece of asbestos felt, moisten with pure wood creosote, campho-phenique, or oil of eucalyptus; cover the side to go next to the pulp with a mixture of iodol, oxid of zinc and vaseline. Now place over the bottom or that portion of the cavity nearest the pulp; now over this place a thin piece of lead or a thin piece of aluminum plate, which will prevent pressure against the bottom of the cavity while inserting either a gold or other metallic filling.

For pyorrhea remove all deposits, and then apply finely pulverized sulfate of copper, which is caustic, astringent and stimulating. It has been my observation for many years that the most aggravated cases of this disease are found in the mouths of those who never eat pickles or anything sour, hence we are in the habit of recommending their use at meals, and also the free use of lemons and oranges. Such patients should be impressed with the importance of adopting an anti-scorbutic diet.

A slip-noose can be put on the lower front teeth with one band, while the rubber-dam is held down with the other; get the slip knots ready first, draw them tight, and they will hold as long as wanted.

In finishing plates always trim the rim low over the bicuspids, leaving it high as can be worn over the cuspids, and the same over and back of the second molars; do not file rim to a knife-like edge, slightly bevel inside of rim at the top, extending down about three-sixteenths of an inch.

To make platinum gold plate, melt with blowpipe pure gold on a piece of platinum, and roll to the desired thickness; the result will be as good as any you can buy.

Gold Solders.—Take a United States $5.00 gold piece, 20 grains coin silver, 10 grains pure copper, 6 grains English toilet pins, melt the silver and copper together first; after melting this and the gold together, add the pins, flow into an ingot and roll, cut it into small pieces and melt again if it should not roll well first time; this will give a solder a little more than nineteen carats fine, and flows nicely on coin gold being the same color.

To solder a cap on a gold tube intended for an artificial crown lay the cap on about a tablespoonful of finely cut asbestos, put the tube in place on the cap, drop in the solder and a little powdered borax, then blow a yellow flame all around the tube till the solder flows, and there will be no danger of melting the plate.

To keep instruments polished, use a material sold in wholesale jewelry houses under the name of diamantine; the method of using is to place a small quantity of the powder on a piece of thick spongy sole leather, and rub the instrument on it, when it will soon take on a fine polish. This diamantine is oxid of zinc, and can be bought in a wholesale drug house much cheaper.

To keep the rubber-dam from jumping off a lower molar tooth, tie a small bead in the thread to be used as a ligature and apply around the tooth so that the bead will come on the lingual side of the tooth, and thus frequently avoid the use of a clamp, which is not admired by many patients.

To take an accurate impression of the mouth for a partial upper set of teeth, smear plaster over the roof of the mouth with the finger, take a string about one foot in length, tie the ends together, put the tied end of the loop into the plaster on the roof of the mouth, and add more plaster to thoroughly imbed the knot, leaving the loop of the string hanging down. In placing the plaster in the mouth care should be taken to have it come full half way over the grinding surfaces of molars and bicuspids, and cutting edges of the front teeth; then trim the plaster and varnish the trimmed surfaces. The plaster should be so trimmed that it will fill up fully one-half of all spaces between the teeth; then cover all the remaining surface of the mouth and teeth with plaster, being very careful to have the teeth well covered and spaces filled in, putting on plaster for the buccal and labial surfaces. When set, the plaster impression readily parts where it has been varnished, the palatal portion is dislodged with the help of the string used, and the pieces are then placed together and the model made. If a tooth is irregular use modeling compound about it and trim suitably; then apply the plaster. When removing it breaks where joined; then remove compound, place in position in the impression and pour the model.

In vulcanite work the best results may be obtained by making models one-fourth marble dust, and three-fourths plaster; also same in flasking the case. The best plaster that I have ever used is that made by Mr. Higginson, at Newburg, N. Y. The best and sharpest models are made without using varnish of any kind on the plaster impression; instead, lather the impression with soap, which is readily done by wetting a camel's-hair pencil and rubbing on a cake of soap and applying to the impression till it is covered, and then set it in a basin of water for a few minutes till the plaster has absorbed all the water it will take, when it should be held under the hydrant for about a minute to wash off the excess of soap; it is now ready for pouring or filling with plaster and marble dust to make the model, which, when it has set sufficiently, can with the exercise of a little care be separated as nicely as if a thick coating of varnish had been used. In this way a sharp model can be made without obliterating any of the fine lines of the impression, which is one of the little things that should always be attended to at the proper time.

In all operations in dentistry it always pays well to attend to the little things at the proper time.—*Review.*

